# The best time for surgery on a patient with recurrent pneumothorax and undetectable culprit lesions is at the exact time air leakage is discovered: a case report

**DOI:** 10.1186/s13019-016-0514-z

**Published:** 2016-08-02

**Authors:** Yousuke Matsumoto, Yoshinobu Hata, Takashi Makino, Satoshi Koezuka, Hajime Otsuka, Keishi Sugino, Kazutoshi Isobe, Sakae Homma, Akira Iyoda

**Affiliations:** 1 Division of Chest Surgery, Toho University School of Medicine, Tokyo, Japan; 2 Division of Respiratory Medicine, Toho University School of Medicine, Tokyo, Japan

**Keywords:** Recurrent pneumothorax, Video-assisted thoracoscopic surgery, Submersion test

## Abstract

**Background:**

One cause of recurrent spontaneous pneumothorax includes overlooking bullae during a previous surgery for pneumothorax; and the identification of the culprit lesions is necessary for prevention of recurrence.

**Case presentation:**

A 28-year-old man was referred to our hospital because of spontaneous right-sided pneumothorax. He underwent video-assisted thoracoscopic surgery, which did not reveal air leakage. The patient was subsequently seen at our hospital for 2 additional episodes of recurrent right-sided pneumothorax. At the third admission we observed intermittent air leakage while the patient was in the sitting position after chest drainage, and we performed surgery. An intraoperative submersion test showed air leakage dorsally from the pleural surface of S^6^ and a minute culprit lesion, which were not seen at the first operation and confirmed the leakage site. The area was ligated and coated with regenerated oxidized cellulose mesh and autologous blood.

**Conclusion:**

In cases of pneumothorax with repeated recurrence, the best time to perform surgery on the patient with undetectable culprit lesion is the exact time that air leakage is observed.

## Background

Video-assisted thoracoscopic surgery (VATS) has become the standard operative approach for treating spontaneous pneumothorax, because it is less invasive than open thoracotomy. However, the recurrence rate of pneumothorax after VATS is significantly higher [1]. One cause of recurrent spontaneous pneumothorax is failure to identify bullae during a previous surgery for pneumothorax; identification of the bullae is necessary for prevention of recurrence. Herein, we describe a patient with recurrent pneumothorax, for whom we were able to use an intraoperative submersion test to identify a minute culprit lesion, because we performed surgery at the time air leakage was observed.

## Case presentation

**Fig. 1:**
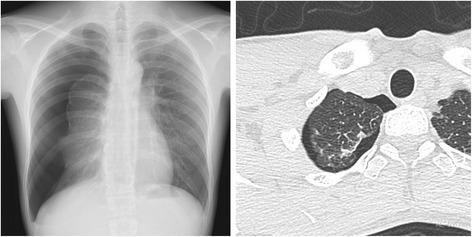
**a** Chest X-ray at the initial presentation revealed a right-sided pneumothorax. **b** Chest computed tomography demonstrated a few tiny blebs on the apex

**Fig. 2:**
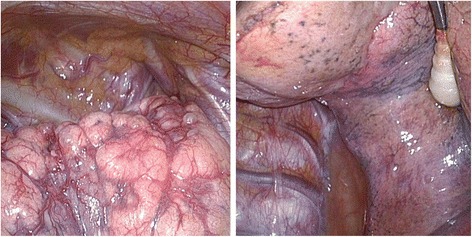
**a** During the first procedure, thoracosopic examination revealed tiny blebs on the apex without obvious air leakage. **b** No pleural changes were seen on the surface of S^6^

A 28-year-old man was referred to our hospital because of right-sided spontaneous pneumothorax (Fig. 1). He was an active smoker with a 10-year history of smoking 40 cigarettes a day, but otherwise his medical history was unremarkable. A chest drainage tube was inserted. After re-expansion of the lung, active air leakage was not apparent. The right lung collapsed with the drainage tube clamped, and VATS was performed. Thoracoscopic examination revealed a few small bullae at the apex of the right upper pulmonary lobe (Fig. 2). An intraoperative submersion test did not reveal active air leakage. Wedge resection was performed for the small bullae in the right upper lobe. The staple line was ligated at both ends and covered with regenerated oxidized cellulose mesh and autologous blood.

Three months later, the patient was referred to our hospital for recurrent right-sided pneumothorax. The air leak disappeared after chest drainage was performed, and an initial clamp test showed collapse of the lung. However, a second clamp test did not result in collapsed lung, and the patient was discharged.

**Fig. 3:**
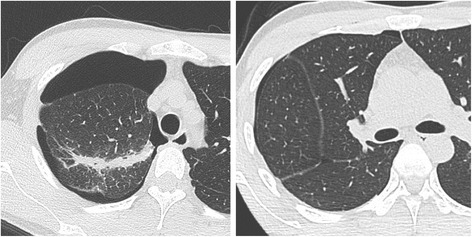
**a** Before the second procedure, chest computed tomography did not show any newly developed bullae near the suture line. (b) No blebs were apparent on the surface of S^6^

**Fig. 4:**
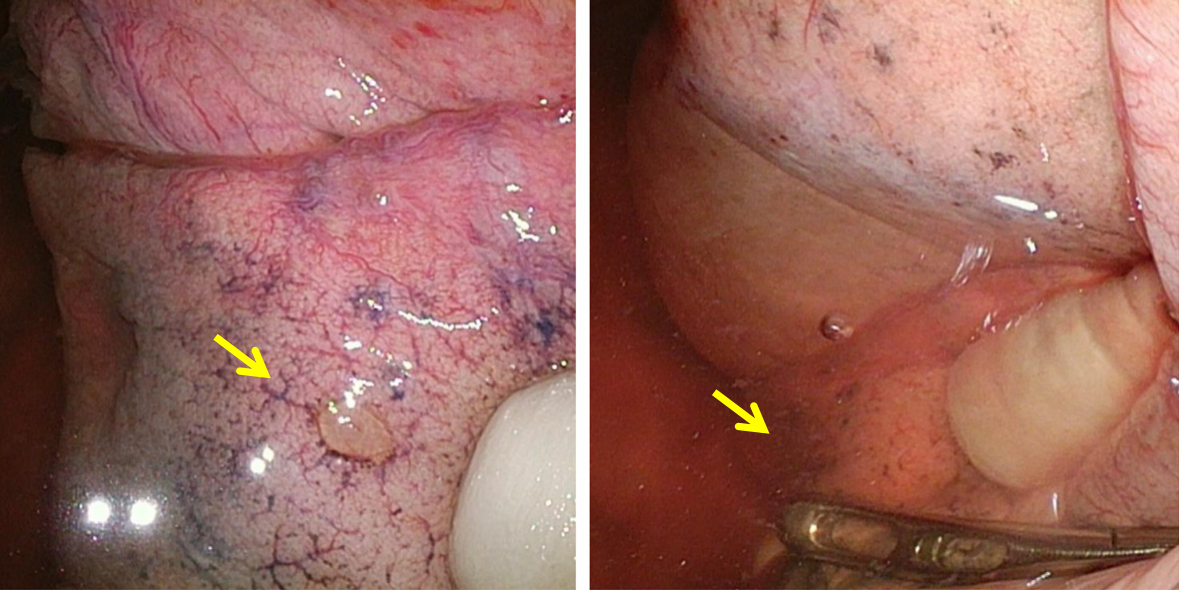
**a** During the second procedure, very small pleural changes with scant air leakage were observed on the surface of S^6^ (arrow). **b** A repeated submersion test revealed the scant air leakage from the S^6^ lesion (arrow)

One month after the second visit, the patient was again referred to our hospital for recurrent right-sided pneumothorax (Fig. 3). Because intermittent air leakage was noticed with the patient in a seated position after chest drainage, we performed surgery. An intraoperative submersion test revealed air leakage dorsally from the S^6^ pleural surface and a minute culprit lesion (Fig. 4), which were not seen during the first operation, confirmed the site of leakage. The affected area was ligated and coated with regenerated oxidized cellulose mesh and autologous blood. The surgery was performed immediately, at the time that air leakage was noted, and we were able to identify the culprit lesion by examining the entire lung in a study to water-seal.

## Discussion

The recurrence rate of pneumothorax is significantly higher following VATS than after open thoracotomy, and has been reported to range from 4.1% to 11.5% [1]. Newly developed bullae around the suture line and failure to identify bullae during surgery are thought to be causes of recurrent pneumothorax. Kurihara et al. reported that regenerated bullae near the suture line were thought to result from the lung being picked up with a stapler deeply and powerfully during thoracoscopic surgery [2]. Therefore, reinforcement of the visceral pleura around the staple line is used in clinical practice [3].

Failure to discover bullae due to collapse of the lung under differential lung ventilation is thought to be an important factor associated with recurrence, and some studies have reported that the recurrence rate of pneumothorax without bullae and blebs was greater than the recurrence rate of pneumothorax with bullae and blebs [4]. The working space of VATS is limited, and therefore to perform an adequate submersion test, the lung parenchyma must be placed in an unnatural position [5]. In our case, we could not identify a bulla at reoperation, but were able to identify a bulla in S^6^ by carefully performing a submersion test of the entire lung. The mediastinal aspects of the lungs in particular are difficult to visualize and are areas where bullae can be overlooked. When the submersion test was negative, the ventilator test procedure was suggested as an alternative [5]. If there is an air-leakage >5% measured by the inhaled/exhaled tidal volume ratio, repeated submersion test was recommended to re-check the leakage.

The indications for surgery for patients with pneumothorax include recurrent pneumothorax, bilateral pneumothoraces, persistent air leakage after drainage, and hemopneumothorax [6]. In addition, from this case, it was thought to become easy to identify a leak point when an operation was performed for the time with the air leak.

## Conclusion

In cases of pneumothorax with repeated recurrence, the best time to perform surgery on the patient with undetectable culprit lesions is the exact time that air leakage is observed.

## Abbreviations

VATS, video-assisted thoracoscopic surgery
